# Effects of single-trial averaging on spatial extent of brain activation detected by fMRI are subject and task dependent

**DOI:** 10.2349/biij.2.3.e27

**Published:** 2006-07-01

**Authors:** SY Fang, JJ Wang, YY Hsu, YL Wan, YY Wai, HL Liu

**Affiliations:** 1 MRI Center, Chang Gung Memorial Hospital, Taoyuan, Taiwan;; 2 Department of Psychology, University of Connecticut, Connecticut, USA;; 3 Department of Medical Imaging and Radiological Sciences, Chang Gung University, Taoyuan, Taiwan;; 4 Department of Medical Imaging, Buddhist Tzu Chi General Hospital, Taipei, Taiwan

**Keywords:** Spatial extent, event-related fMRI, trial averaging

## Abstract

**Aim:**

The effects of single-trial averaging on the spatial extent of event-related fMRI activation may vary between subjects and tasks. The purpose of this study was to evaluate this variability using a visual task and a word generation task.

**Patients, materials, and methods:**

Five Chinese right-handed male volunteers participated in the experiment. Experiments were conducted using a 1.5 T clinical MRI scanner with a T2*-weighted single-shot gradient-echo EPI sequence. Each task contained 150 trials that were separated into 5 runs. For each voxel, time courses averaged across different numbers of randomly selected trials, were obtained. They were applied for determining the voxels with significant activations, using a students’ t-test (p<0.001, uncorrected).

**Results:**

Consistent with previous findings, the number of the activated voxels increased monotonically with the number of trials combined. The ascending rate and the maximum number of the activated voxels were different, however, between tasks and among subjects.

**Conclusions:**

The effects of single-trial averaging were found to vary significantly between tasks and subjects. Therefore, we strongly advise to carefully consider such variability when using the spatial extent of activation as a measure in a group or a task comparison.

## INTRODUCTION

The spatial extent of the activation is a critical measure in many fMRI studies [1-3], and the differences may be interpreted as hypoactive or dysfunctional. In addition, the size of the spatial extent can be used to reduce the detection of the false activation [4]. The true regions of activation tend to occur over contiguous voxels, where noise showed much less tendency to form clusters [5]. Few studies have, however, directly addressed those issues related to the spatial extent of the BOLD response.

One previous study found that an exponential relationship existed between the number of trials and the spatial extent. Over 100 trials were averaged in this event-related study, using a visual stimulation task [6]. Saad *et al.* also reported the spatial extent increased monotonically with trial averaging in a block-designed visual task [7].

Recently, event-related fMRI has been widely applied in the exploration of human cognitive process [8]. The shape of the hemodynamic response function, however, varies between areas in the visual and the motor cortices even in the same subject [9]. Therefore, the effect on the spatial extents in averaged trials would remain unclear in different brain areas using different stimuli, such as, cognitive tasks. The optimised number of trials for the steady-state response in a cognitive experiment using event-related fMRI design requires further investigation.

In this study, a cognitive task using word generation [10] was designed to examine the effects of single-trial averaging. The result was compared with that from a visual task. Different effects between both tasks will be discussed and an optimised value of averaging trials will be considered for the future applications. It should be noted that the spatial extents detected by fMRI can be interpreted as involvement of neural activity only if the steady-state condition in single-trial averaging is reached, i.e., independent on contrast-to-noise ratio (CNR). Therefore, the current study is crucial for the experiments studying graded activations, as well as clinical fMRI where CNR of the hemodynamic responses in patients can be quite different.

## MATERIALS AND METHODS

The experiments were conducted at the MRI Centre, Chang Gung Memorial Hospital, LinKou, Taiwan. Five male volunteers participated in this study. They were aged 20-26 years, with a mean age of 22.4 years. They were native Chinese speakers. The participants had no history of neurological or psychiatric disorders and reported to be right handed, which was confirmed with the Handiness inventory [11]. A written, informed consent was obtained in all cases.

Neural activation was monitored by a 1.5 T Magnetom Vision MRI scanner (Siemens, Erlangen, Germany) with a single-shot gradient-echo EPI sequence (TR / TE /FA = 1000 ms / 60 ms / 90°, Slice Thickness = 8 mm, in-plane resolution = 3.3 mm). The stimuli were shown through a goggle display system (Resonance Technology Inc., CA, USA). Corrective lenses were used when necessary. Prior to the MR imaging, each subject was visually familiarised with the procedures and the experimental conditions, to minimise anxiety and enhance task performance. The subject, before being transferred to the scanner, was fitted with a plastic earmuff and a tightly fitting, thermally moulded, plastic facial mask that extended from the hairline to the chin.

**Figure 2:**
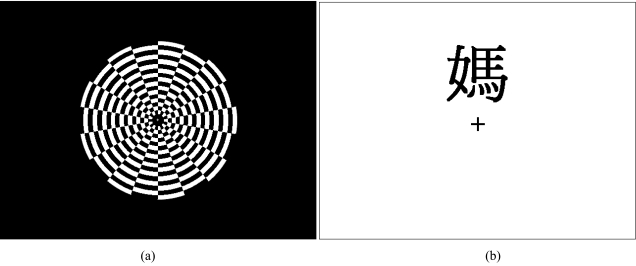
An example of the slide used in the a) visual task; and b) word generation task.

Subjects participated in both event-related fMRI experiments, a visual stimulus and a word generation task, respectively, on two separate days. This was done to reduce subject discomfort in a prolonged scanning session. The time span between both experiments was set at one week. The experiment consisted of five separate runs of total 150 trials. Each run was made of 460 s, starting with 10-s dummy scans and followed by 30 trials (0.5 s stimulus + 14.5 s fixation). The visual task was a black and white circular checkerboard flashing at 8Hz (Figure 2a). Seven contiguous slices were acquired parallel to the calcarine sulcus. In the word generation task a different, single Chinese character was displayed each time (Figure 2b), and the subject was asked to silently associate it with a semantically related word. All characters used were sampled from the Mandarin Promotion Council (Ministry of Education, Taiwan), with a frequency ranging between 15 and 25 per 10 million occurrences. Seven contiguous slices were acquired parallel to the AC-PC line.

The data analyses for each subject were performed within an anatomically defined region of interest (ROI) within the middle slice. ROIs were chosen, based on the anatomical images, in the gray matter along the calcarine fissure in the primary visual cortex in visual tasks or at BA10, 44, 45, and 47 in word generation tasks [12]. All analyses were conducted in Matlab (The Math Works, Inc., Natick, MA, USA). The time-serial data were normalised to the averaged signal intensity of the 1st, 2nd, and 15th time points within each single trial. The single trial populations were sampled randomly. A series of averaged signals, varying in the number of trials combined, were then computed for each subject. Twenty averaged signals were computed for each possible number of trials combined. For each averaged signal, a Pearson product-moment correlation coefficient was calculated between the mean time course and a gamma variate function. Activated voxels were determined using a student’s t-test (t >3.8, p < 0.001, uncorrected). The number of voxels that exceeded the threshold was determined for each of the twenty averaged signals, at each number of combined trials for each subject.

## RESULTS

**Figure 1:**
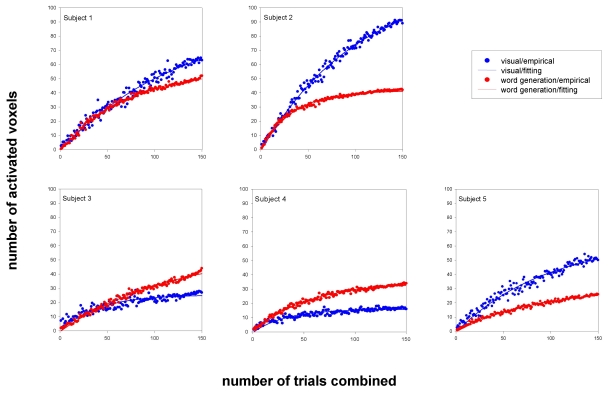
The number of activated voxels plotted against the number of trials combined from all subjects.

Figure 1 shows, from all five subjects, the number of the activated voxels plotted against the number of trials combined. Consistent with previous studies, the volume of activation increased with the number of trials combined in the visual task as well as the word generation task, in all subjects in this experiment. The number of activated voxels at x trials combined, V_x_ , was then fitted with an exponential function:

(1) $$V_{x}=V_{\max}\left[1-\exp\left(-x/\xi \right)\right]$$

where V_max_ represented the number of total expected activated voxels and ξ, the ascending rate, equalled to the number of trials combined when V_x_ is equal to 63% of V_max_. Table 1 showed the V_max_ and ξ from all subjects in both experiments, respectively. The ascending rate and the number of total expected activated voxels varied significantly between tasks and among subjects.

**Table 1:** The number of total expected activated voxels, V_max_, and the ascending rate, ξ, from all subjects in both visual stimulation and work generation experiments.

**Subject**	**Visual stimulation**			**Word generation**	
	V_max_	ξ		V_max_	ξ
1	92.25	126.38		56.32	69.51
2	120.51	104.52		41.32	32.65
3	25.30	38.74		57.39	122.85
4	15.89	32.17		34.58	52.07
5	64.32	95.68		30.23	83.47

## DISCUSSION AND CONCLUSION

Our findings are consistent with the observations of Huettel and McCarthy [6]. The spatial extent of the fMRI activation in a visual task is influenced by the number of trials combined. Similar results were observed in a word generation task, which indicated that the spatial extent increased with the number of trials combined in an event-related fMRI experiment; despite the different tasks performed or brain areas studied.

Figure 1 shows that the number of the activated voxels did not reach the plateau even after averaging 150 trials. Table 1 shows that the maximum number of the detected active voxels is, however, close to V_max_ in each subject. This suggests that most of the activated voxels were identified and is consistent with the report of Huettel and MaCarthy [6].

Our experiment showed that the effects of single-trial averaging on the spatial extent of an event-related fMRI study may vary between tasks and among subjects. The spatial extent can be affected by the amplitude of the hemodynamic response [13] or the voxel-wise noise. Our results indicate that such effects should be taken into consideration in experiments using different tasks and involving different brain areas or in a comparative study on subjects. Similar findings were reported by Duann *et al.* [14], who demonstrated that the hemodynamic response varied substantially across trials as well as sessions, subjects, and brain areas. Purdon *et al.* [15] concluded that there exists a wide range of noise variances in the BOLD signal within and between subjects, which could affect the determination of the spatial extent.

The wide range of noise variances might have led to the different ξ between tasks and subjects in our experiment. An optimised number of trials have to be carefully considered in future applications, to reduce the noise. The variances in V_max_ in this study can be explained by the between-subjects reproducibility effect as was reported by the previous studies [16, 17]. From the current study and the within-subject reproducibility of the spatial extent [16, 18-21], the use of the spatial extent of the activation as an index to compare the results from different groups and/or tasks could introduce errors. Furthermore, the use of spatial extent as a measure of activation could be a source of variance in itself [22].

To conclude, ξ, the ascending rate of the detected activation volume and V_max_, the number of total expected activated voxels vary across different tasks and subjects when increasing number of trials are combined. This may be due to the complex contrast and noise characteristics from the various effects in the hemodynamic changes in different task performances, brain areas, and other subject-dependent physiological parameters. Special precautions should be taken in fMRI studies, when using detected spatial extent as an index for quantitative comparisons.
